# Blood-Brain Barrier Deterioration and Hippocampal Gene Expression in Polymicrobial Sepsis: An Evaluation of Endothelial MyD88 and the Vagus Nerve

**DOI:** 10.1371/journal.pone.0144215

**Published:** 2016-01-20

**Authors:** Gerard Honig, Simone Mader, Huiyi Chen, Amit Porat, Mahendar Ochani, Ping Wang, Bruce T. Volpe, Betty Diamond

**Affiliations:** 1 Center for Autoimmune and Musculoskeletal Diseases, Feinstein Institute for Medical Research, Manhasset, New York, United States of America; 2 Center for Translational Research, Feinstein Institute for Medical Research, Manhasset, New York, United States of America; 3 Laboratory of Biomedical Science, Feinstein Institute for Medical Research, Manhasset, New York, United States of America; Texas A&M University Health Science Center College of Medicine & Baylor Scott and White Health, UNITED STATES

## Abstract

Systemic infection can initiate or exacerbate central nervous system (CNS) pathology, even in the absence of overt invasion of bacteria into the CNS. Recent epidemiological studies have demonstrated that human survivors of sepsis have an increased risk of long-term neurocognitive decline. There is thus a need for improved understanding of the physiological mechanisms whereby acute sepsis affects the CNS. In particular, MyD88-dependent activation of brain microvascular endothelial cells and a resulting loss of blood-brain barrier integrity have been proposed to play an important role in the effects of systemic inflammation on the CNS. Signaling through the vagus nerve has also been considered to be an important component of CNS responses to systemic infection. Here, we demonstrate that blood-brain barrier permeabilization and hippocampal transcriptional responses during polymicrobial sepsis occur even in the absence of MyD88-dependent signaling in cerebrovascular endothelial cells. We further demonstrate that these transcriptional responses can occur without vagus nerve input. These results suggest that redundant signals mediate CNS responses in sepsis. Either endothelial or vagus nerve activation may be individually sufficient to transmit systemic inflammation to the central nervous system. Transcriptional activation in the forebrain in sepsis may be mediated by MyD88-independent endothelial mechanisms or by non-vagal neuronal pathways.

## Introduction

Sepsis is a devastating syndrome in which a physiological stimulus, usually a blood-borne infection, triggers a potent systemic inflammatory state which leads to multi-organ dysfunction. Sepsis is a leading cause of death and disability throughout the world [1], with premature infants and elderly patients most vulnerable. Sepsis incidence has been rising steadily in the United States, likely due to the aging population. Despite the implementation of clinical guidelines for diagnosis and symptom management [2, 3], patients who survive an acute episode of severe sepsis are at increased risk for disability and death due to dysfunction in immunity, cognition and other domains [4–7].

While the pathophysiology of sepsis is not fully understood and is likely to be in part organ-specific [8, 9], convergent evidence from clinical observations and experimentation in animal models indicates that stimulation of molecular pattern receptors [10], induction of localized and circulating cytokines [11] and loss of microvascular integrity [12] are general mechanisms. Sepsis is characterized by the activation of the myeloid cells of innate immune system and other cell types, including endothelial cells, primarily through the Toll-Like Receptor (TLR) molecular pattern recognition pathway [10, 11]. This stimulation results in the secretion of successive waves of cytokines into the circulation (a “cytokine storm”) [13]. The mobilization of this overwhelming innate immune response may contribute to resolving the initial insult (tissue injury or infection) but can itself also lead to tissue damage, resulting in the release of additional inflammatory mediators and creating a dangerous positive feedback cycle [13]. In particular, the resulting loss of microvascular integrity is thought to be a central feature of sepsis pathophysiology across multiple organs [12].

The central nervous system (CNS) and its vasculature are responsible for critical physiological functions during sepsis and are also particularly vulnerable to injury under such conditions [14, 15]. In the CNS, the vast majority of endothelial cells exhibit a rigid blood-brain barrier (BBB), preventing the diffusion into the CNS of polar soluble factors (ions, peptides, proteins, antibodies etc.) [16, 17]. During systemic infection, the luminal surfaces of cerebrovascular cells are exposed to a complex set of physiological stimuli including pathogen-associated molecular patterns (PAMPs), endogenous danger-associated molecular patterns (DAMPs), cytokines, chemokines, and altered blood pressure. These stimuli lead to alterations in CNS vascular physiology (such as increased blood-brain barrier permeability) and short- and long-term CNS-intrinsic inflammation. With the deterioration of the BBB, molecules may penetrate into the CNS which may be toxic and/or which may communicate the presence of systemic infection to the CNS, even without overt CNS infection [18]. This deterioration of BBB function may be critical for sepsis-induced neuropathology, as circulating signals including (but not limited to) cytokines can disrupt CNS homeostasis even at concentrations much lower than in the circulation of a septic individual [17, 19–24]. Importantly, it has been demonstrated *in vivo* that the functions of brain microvascular endothelial cells and the BBB are responsive to systemic inflammation as well as to inflammation within the CNS [15, 22, 25–30].

At the same time, blood-to-brain signaling at the vascular interface is thought to be an important mechanism whereby the CNS integrates information to orchestrate adaptive responses [31–36]. In fact, the CNS mediates important reflexes during sepsis, such as fever [37] and modulation of systemic cytokine secretion [38]; such responses are thought to involve vascular signaling as well as neuronal signals from the autonomic nervous system [18]. Acute neurovascular inflammation and resulting BBB compromise may thus be required for the integration of blood-borne signals by the CNS and may trigger adaptive and maladaptive CNS responses. These may include neuroinflammation in sepsis with long-term cognitive impairment, described in animal models as well as in patients [4, 5, 39–41], and the proposed potentiation of neurodegenerative processes following systemic infection [42–45]. However, a mechanistic understanding of BBB function during sepsis remains incomplete.

Most studies addressing these issues have employed endotoxemia models in which experimental animals are treated with high concentrations of purified microbial components to stimulate innate immune responses. Widely used stimuli include lipopolysaccharides (LPS), which activate the TLR pathway primarily through the TLR4 receptor, and cytokines implicated in sepsis, such as interleukin 1β (IL-1β) [18, 32, 34, 46–50]. Such studies have led to the conclusion that TLR signaling in endothelial cells is required for CNS transcriptional and physiological responses to systemic inflammation [28, 29, 48, 49, 51, 52]. Although these models have generated many important insights into the biology and pathophysiology of innate immunity, important mechanistic differences are thought to exist between endotoxemia and live infection or sepsis. In particular, polymicrobial sepsis, in which a combination of bacterial pathogens (often originating from a breach in the gut barrier) infects the blood, presents a much more complex set of microbial patterns and stimulates a multiphasic cytokine storm which is distinct from that elicited by endotoxemia [53]. In order to simulate this situation, several experimental models have been developed. The cecal-ligation and puncture (CLP) model of acute peritonitis, in which sepsis is triggered by surgical perforation of the bowel, represents a particularly well-studied model which is thought to recapitulate the many of the canonical features and dynamics of severe polymicrobial sepsis in patients [54–56]. Although several studies have demonstrated physiological responses to CLP in the CNS, few have addressed the role of vascular endothelial cells or of autonomic neuronal signaling in such responses.

In this study, we used two-photon *in vivo* imaging in the murine CLP model of polymicrobial sepsis to directly visualize physiological effects of sepsis on CNS vasculature. We further characterized transcriptional responses to CLP by quantifying the expression of key markers of inflammation in the hippocampus region of the CNS. The hippocampus was chosen as previous studies have established this region is susceptible to sepsis-induced neuropathology [41, 57]. In order to interrogate the role of TLR signaling in endothelial cells, we performed these analyses in mice lacking endothelial expression of MyD88, an adaptor protein important for canonical TLR signaling. Finally, we assessed the requirement of the vagus nerve in hippocampal transcriptional activation following CLP.

## Results

In order to evaluate the potential role of endothelial cells in CNS manifestations of sepsis, we chose to abrogate MyD88-dependent signaling in endothelial cells. MyD88 is responsible for most canonical TLR signaling, such as TLR4-mediated sensing of LPS, as well as for canonical IL-1 signaling [58, 59], and is thus broadly required for multiple cellular responses during polymicrobial sepsis [60].

**Fig 1:**
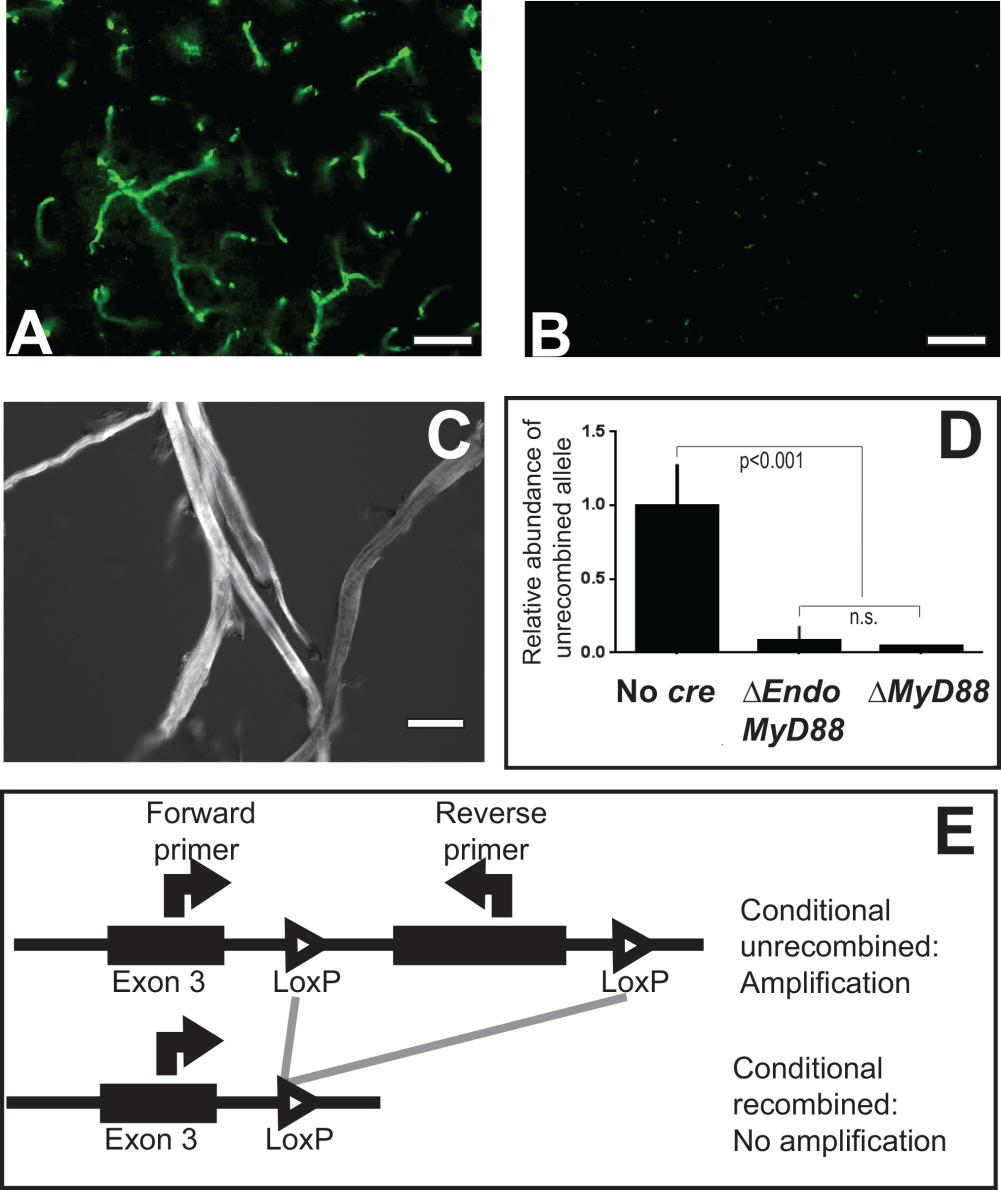
Specific and efficient endothelial-specific deletion of *myd88* in CNS endothelial cells. A transgene driving the expression of *cre* recombinase under the control of the *tek* promoter was used to target endothelial cells. **A**: Histological detection of GFP expression in a specific vascular pattern in hippocampus sections from ROSA26 reporter mice carrying the *tek-cre* transgene. **B**: No recombination was evident in ROSA26 single transgenic mice in the absence of the *cre* transgene. **C**: Phase contrast image of CNS microvascular fragments purified using a density gradient method. **D**: qPCR for the unrecombined *myd88* allele in hippocampal microvascular DNA from *myd88*^Flox/Flox^ (No *cre*), ΔEndoMyD88 and constitutive deletion (ΔMyD88) mice. Note the near-complete deletion of *myd88* in microvascular DNA from ΔEndoMyD88 mice (p<0.001, t-test). Sample size, 3 per genotype. Error bars represent standard error of the mean. Scale bars, 50 μm (A, B); 10 μm (C). **E:** Diagram of genomic primer binding sites for assays used to generate data in D.

In order to assess the specific role of endothelial TLR signaling in the CNS, we generated mice lacking a functional *myd88* gene in the endothelial cell lineage (ΔEndoMyD88 mice) using the *cre-LoxP* system. Cre recombinase expression was directed with a hemizygous transgene containing a proximal 5’ regulatory region from the *tek* gene locus region, as described [61]. The conditional *myd88* allele used here, with *loxP* sites flanking a critical exon, has also been described. Successful Cre-mediated excision of exon 3 results in a frame-shifted null allele, with complete loss of functional MyD88 protein (Fig 1E) [62]. Importantly, this conditional mutant allele has been previously shown to generate endothelial-specific *myd88* null mutant mice, in combination with a transgenic mouse strain expressing Cre recombinase directed by the *tek* regulatory region [63], as described here.

Both lines were obtained from Jackson Laboratories following extensive backcrossing to the C57BL6/J strain. Male ΔEndoMyD88 mice bearing the *tek-cre* transgene and homozygous for a *loxP*-bearing *myd88* locus (*tek-cre*^Cre/0^; *myd88*^Flox/Flox^) and corresponding control male littermates without the *tek-cre* transgene but homozygous for the conditional *myd88* allele (*tek-cre*^0/0^; *myd88*^Flox/Flox^), designated wild-type (WT), were bred in-house from a single colony and analyzed between 3 and 5 months of age. In order to validate the specificity of recombinase expression in the CNS, mice bearing the *tek-cre* transgene were interbred with a reporter mouse strain, bearing a Cre-activated Green Fluorescent Protein (GFP) transgene at the ROSA26 locus. Resulting *tek-cre*^Cre/0^; *rosa26*^GFP/+^ mice exhibited CNS GFP expression in a characteristic vascular pattern (Fig 1A), whereas no GFP expression was observed in single transgenic *tek-cre*^0/0^; *rosa26*^GFP/+^ mice in the absence of the *tek-cre* transgene (Fig 1B).

In order to test whether *myd88* was efficiently deleted in CNS endothelial cells, we quantified the level of the intact allele in purified CNS vasculature in ΔEndoMyD88 mice; *tek-cre*^0/0^; *myd88*^Flox/Flox^ mice (WT or ‘No c*re*’); and homozygous *myd88* null mutant mice, lacking functional MyD88 in all cells (ΔMyD88) [64]. We purified microvascular fragments from CNS tissue from mice of all three genotypes, using a density gradient technique resulting in a highly pure microvascular fraction, as determined by Nomarski microscopy (Fig 1C). We then prepared genomic DNA from the microvascular fractions. Genomic DNA was subjected to quantitative polymerase chain reaction using an assay which detected the functional unrecombined allele, but not the null recombined allele (Fig 1E). As expected, the functional *myd88* allele was readily detected in WT microvascular genomic DNA, but was undetectable in microvascular DNA from constitutive ΔMyD88 null mutant mice. The levels of the functional *myd88* allele in CNS microvasculature were statistically indistinguishable between ΔEndoMyD88 and ΔMyD88 mice (Fig 1D) (p = 0.6), indicating that ΔEndoMyD88 mice lack the capacity to generate MyD88 protein in the vast majority of microvascular cells.

We next subjected both ΔEndoMyD88 and WT mice to CLP. All analyses were performed at 24 hours post-surgery; at this time point, reduced locomotion, fecal discharge, dehydration, and bacteremia were consistently observed in both groups (data not shown). In previous histological studies in this model, we have not observed evidence of bacterial infection in the CNS parenchyma [41]. For control (sham surgery) groups, cecum manipulation was performed in the absence of ligation and puncture; bacteremia and behavioral signs of distress were consistently absent. Mortality during the 24 hours following CLP was rare (<5%), with no overt effect of genotype; mortality was not observed in sham-operated mice (data not shown).

**Fig 2:**
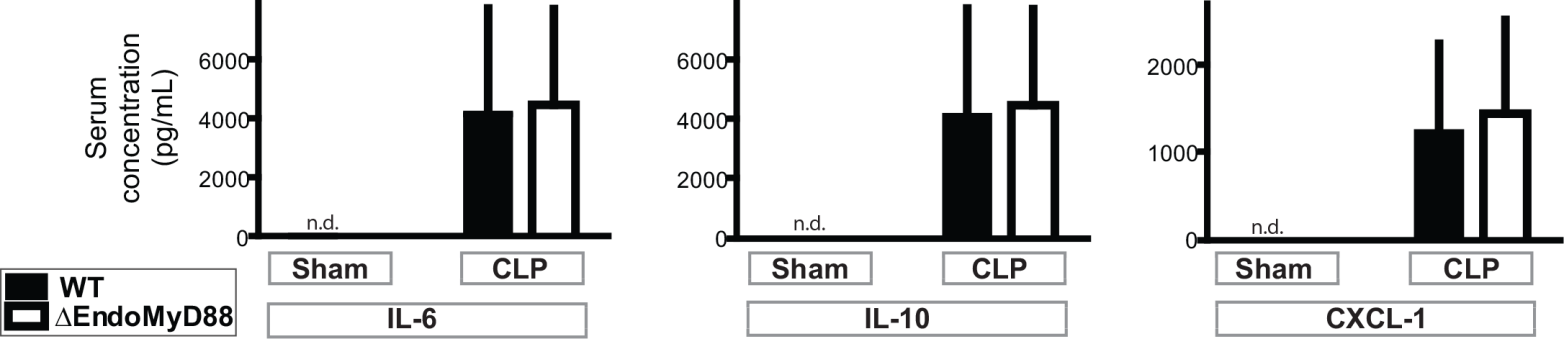
Systemic innate immune response to polymicrobial sepsis in ΔEndoMyD88 mice. CLP or Sham laparotomy was performed in ΔEndoMyD88 mice and littermate controls. 24 hours post-surgery, sera were collected and levels of IL-6, IL-10 and CXCL-1 were quantified. n.d., below the limit of detection. For all 3 analytes, a significant effect of surgery was observed (p<0.001) with no effect of genotype and no interaction (p>0.1) (2-way ANOVA). Sample size, 6–7 per condition. Error bars represent standard error of the mean.

TLR signaling by myeloid cells is important for cytokine secretion during sepsis, and the *tek-cre* transgene used here has been reported to be expressed in a subset of hematopoietic stem cells [61]. In order to determine whether ΔEndoMyD88 mice mounted a comparable systemic response to CLP, we assessed serum levels of IL-6, IL-10 and CXCL-1 in the serum 24 hours after CLP (Fig 2). IL-6 is the most well-established early biomarker of sepsis severity in the murine CLP model, as early levels of IL-6 in the serum are highly predictive of later mortality [65]. IL-10, a negative regulator of inflammation, and CXCL-1, a chemokine involved in neutrophil migration, are also well-characterized serum markers of CLP sepsis severity [56, 66]. These factors were undetectable in sham-operated mice and elevated in septic mice, whereas the increases in these circulating signals were comparable in WT and ΔEndoMyD88 mice (Fig 2). Although the significant variability in circulating cytokines during CLP sepsis may preclude quantitative comparison of systemic inflammation between WT and ΔEndoMyD88 mice, these results suggest that ΔEndoMyD88 mice do not have fundamental impairment in the systemic cytokine response to polymicrobial sepsis.

**Fig 3:**
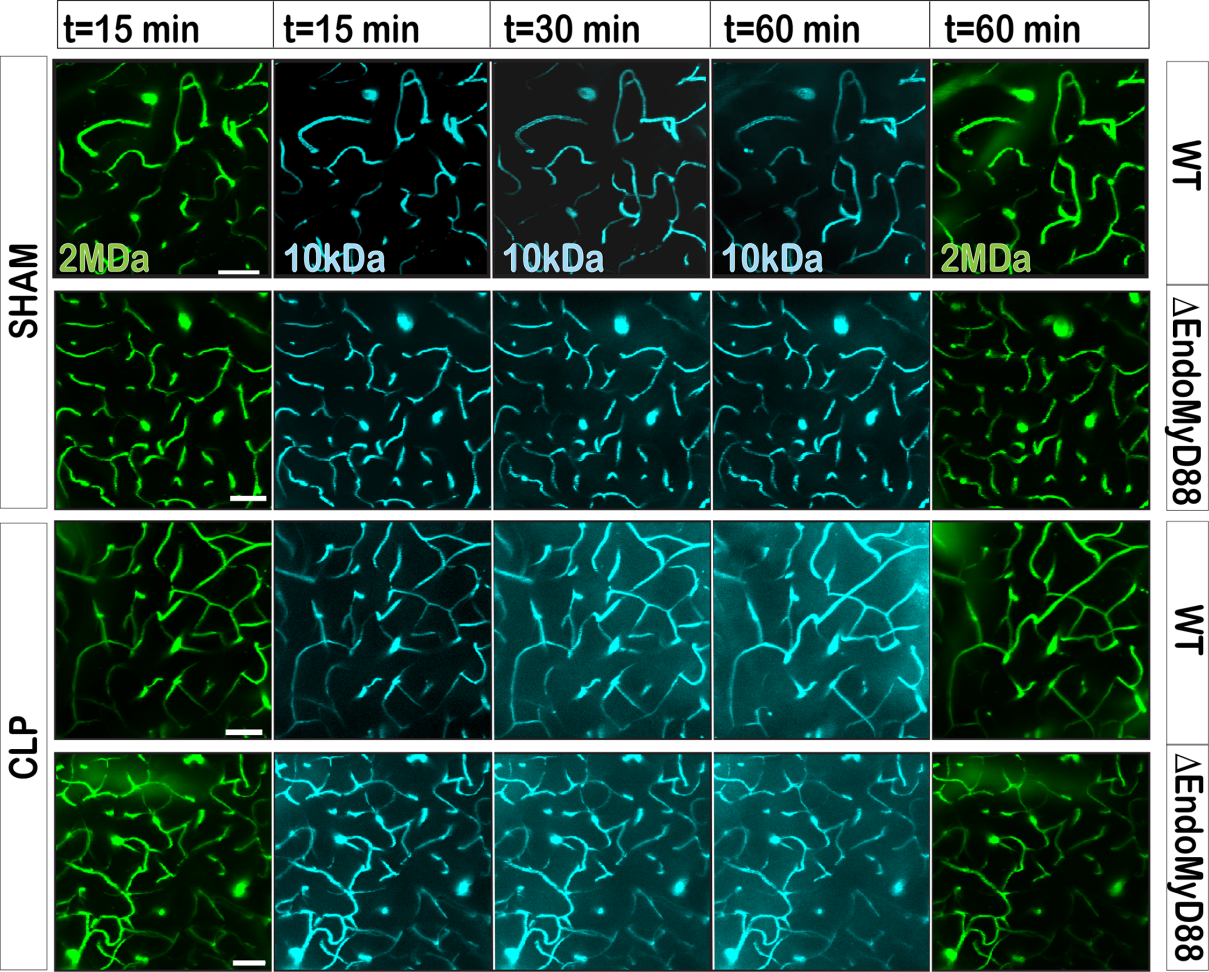
Blood-brain barrier permeabilization during polymicrobial sepsis is independent of endothelial MyD88. CLP or Sham laparotomy was performed in mice of the indicated genotypes. 24 hours later, transcranial 2-photon imaging was performed *in vivo*. At t = 0, two distinctly labeled dextrans of low and high molecular were injected *i*.*v*.: FITC-dextran, 2 MDa, and CascadeBlue-dextran, 10KDa. Imaging was performed over the following 60 min. Images shown are maximal intensity z-stack projections of 30 images acquired in 1 μm steps starting 30–40 μm below the pia. Time denotes the time of initiation of image acquisition. Scale bar, 50 μm.

CLP-induced sepsis has been shown, using a variety of experimental methods, to degrade BBB integrity [19, 21, 23]. This phenomenon is thought to also occur in clinical sepsis [14]. In order to directly visualize CLP-induced deterioration of the BBB, we adapted an *in vivo* 2-photon imaging preparation [67] and analytical method [68]. CLP or sham surgery was performed; 24 hours later, mice were anesthetized for imaging. Imaging was performed using a thinned-skull preparation [67] in order to minimize artifactual anatomical disruption and inflammation resulting from the imaging process. In order to visualize BBB breakdown, we adapted a published method [68] by employing simultaneously a dextran dye of intermediate (10 KDa) and a high (2 MDa) molecular weight dye, using two different labels. Dyes were intravenously injected at time 0 and imaged simultaneously using appropriate emission filters at 15, 30 and 60 minutes post injection (Fig 3). The high molecular weight dye was not expected to extravasate significantly into the CNS parenchyma, even with BBB breakdown [68], providing a reference vascular image. To quantify images, an extravasation index was calculated at 0 and 30 minutes post injection from the image intensity corresponding to extravascular 10 KDa dye (see Methods and Table 1).

**Table 1:** Quantification of BBB permeability by *in vivo* imaging.

Mouse	Genotype	Surgery	Δ[30–0 min]
1	WT	Sham	-1.4
2	ΔEndoMyD88	Sham	-3.9
3	WT	CLP	15.8
4	ΔEndoMyD88	CLP	8.4
5	ΔEndoMyD88	CLP	10.6
6	ΔEndoMyD88	CLP	17.1

Extravasation index was calculated as described in Methods.

We first performed imaging in a sham-operated WT mouse and a sham-operated ΔEndoMyD88 mouse. As expected, the 10 kDa and 2 MDa dyes both remained confined to the vascular lumen throughout the imaging session in both mice (Fig 3 and Table 1). We then performed imaging in a CLP-operated WT mouse, and observed a progressive extravasation of the 10 KDa dye, but not the 2 MDa dye. Three CLP-operated ΔEndoMyD88 were then subjected to imaging; in all three cases, progressive extravasation of the 10 KDa dye was observed (Fig 3 and Table 1). The small sample size precludes specific conclusions regarding the relative extent of BBB degradation in the experimental conditions analyzed. However, the data support that endothelial MyD88 is not required for loss of BBB integrity during CLP sepsis.

In order to determine whether endothelial MyD88 is required for CNS transcriptional responses to sepsis, we assessed the mRNA transcript levels of a panel of genes reported to be highly expressed during CLP sepsis. We used quantitative reverse transcription polymerase chain reaction (qRT-PCR) and focused on the hippocampus, a discrete forebrain structure critical for learning, memory and other cognitive functions, because we have previously demonstrated that CLP causes long-term structural changes in the hippocampus, associated with impaired performance in learning and memory tasks [41]. In order to reduce the potential contribution of circulating immune cells to transcript levels, mice were transcardially perfused with saline prior to sacrifice. We assessed expression of a panel of genes reported to be highly upregulated during CLP sepsis, including *tnfa* (tumor necrosis factor α) and *il1b* (interleukin 1 beta), which were undetectable in the CNS in any condition despite marked upregulation in the liver following CLP (data not shown).

**Fig 4:**
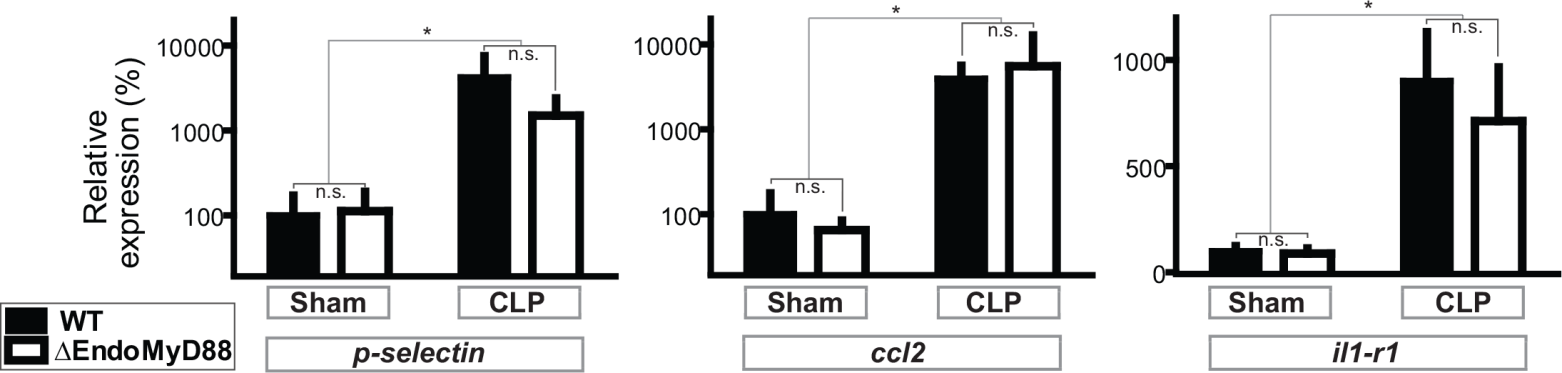
CNS transcriptional response to polymicrobial sepsis do not require endothelial MyD88. ΔEndoMyD88 mice and WT controls were subjected to CLP surgery or Sham laparotomy. Hippocampi were dissected 24 hours post-surgery and expression of indicated genes was analyzed by qPCR. Data were analyzed by 2-way ANOVA. *p<0.001, significant effect of surgery; n.s., p>0.1, no significant effect of genotype and no significant interaction (2-way ANOVA); error bars represent standard error of the mean. Sample size, 5–7 per condition.

In contrast, three transcripts, differentially expressed in endothelial, microglial and neuronal cells, exhibited robust induction following CLP. *Selp* encodes p-selectin, an adhesion molecule expressed primarily by endothelial cells and a classic expression marker of endothelial inflammation [30, 69–71]. *Ccl2* encodes CCL-2, a chemokine which is expressed by endothelial and microglial cells during CNS inflammation [72–74]. *Il1r1* encodes the interleukin-1 receptor type 1, which has been shown to be critical for synaptic plasticity and neuroinflammation [50, 57, 75–77] and is induced in hippocampal neurons during CLP sepsis [57], but which may also be highly expressed in inflamed endothelial cells. Expression levels of *Selp*, *Ccl2* and *Il1r1* were robustly increased by CLP sepsis (Fig 4) irrespective of genotype, suggesting that endothelial MyD88 is not required for transcriptional responses to CLP in hippocampal vasculature, microglia and neurons.

**Fig 5:**
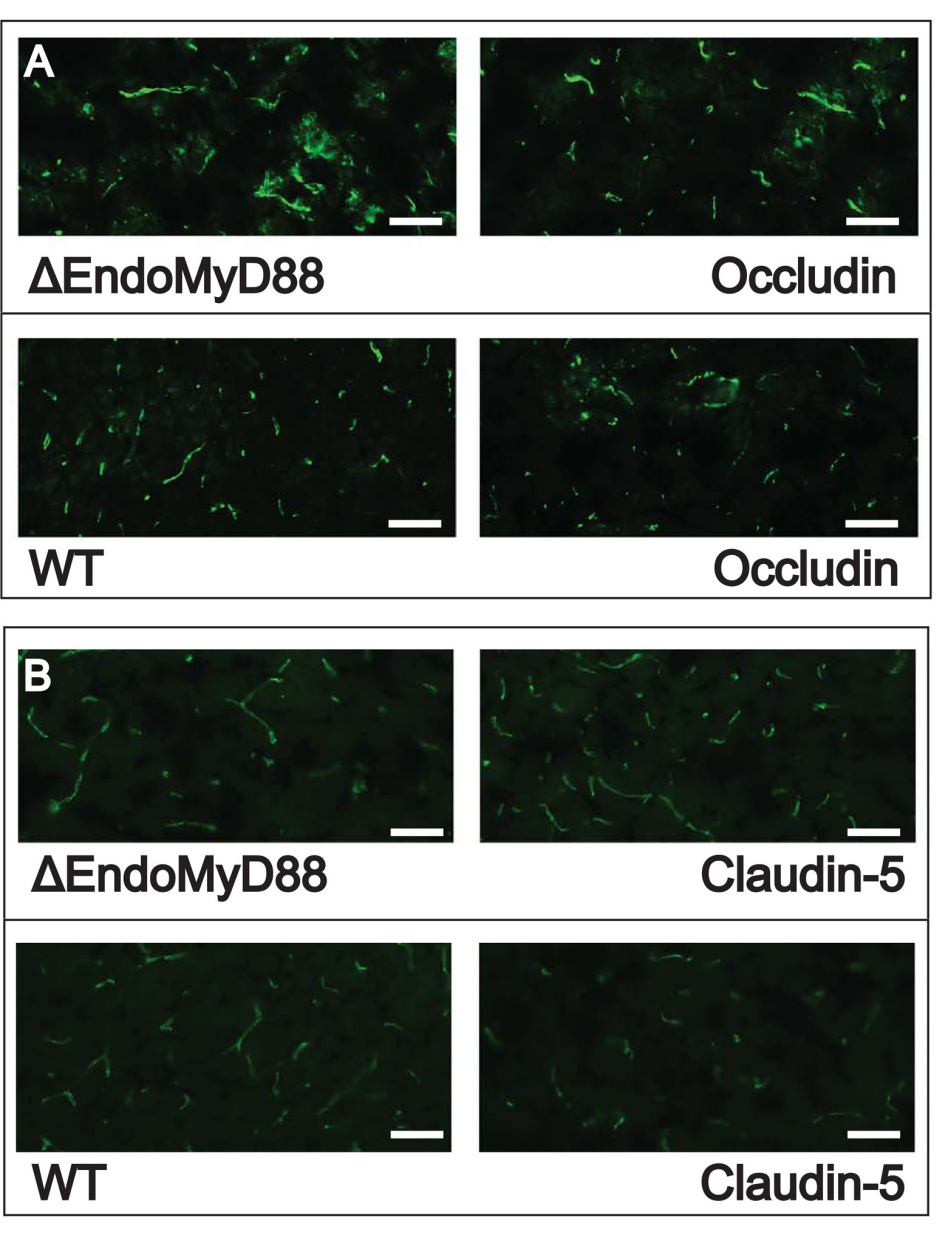
Immunofluorescence staining of BBB proteins in CLP-operated ΔEndoMyD88 mice and WT mice. Brains were dissected from ΔEndoMyD88 mice and WT mice at 24 hours post CLP surgery. Cortical tissue was sectioned, immunostained for occludin (A) or claudin-5 (B) proteins, and imaged using fluorescence microscopy. Images shown are representative sections from two independent mice (n = 3 mice per condition). Scale bar, 50 μm.

**Fig 6:**
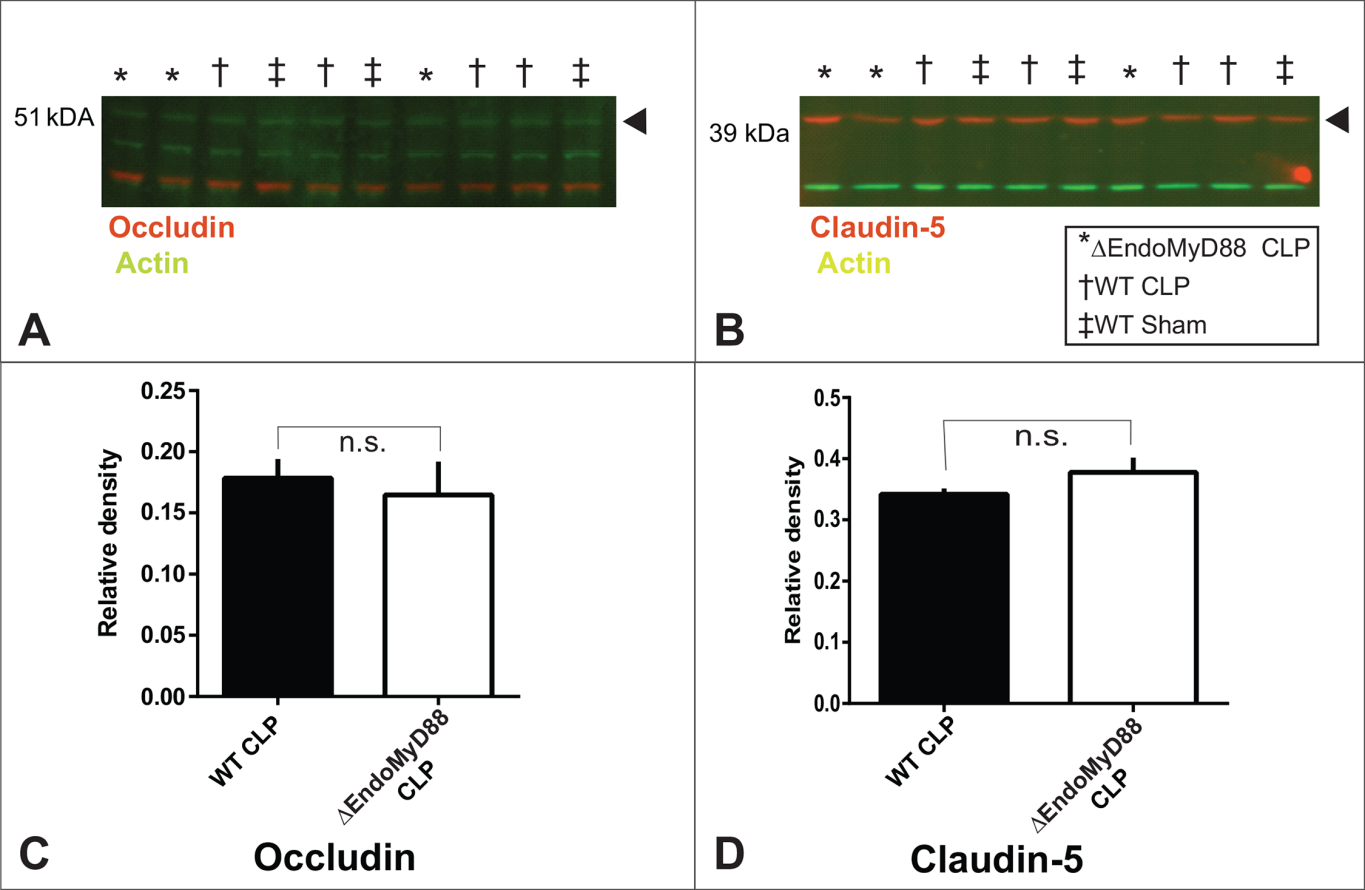
Immunoblot quantification of BBB proteins in CLP-operated ΔEndoMyD88 mice and WT mice. Protein homogenates were prepared from hemibrains dissected from ΔEndoMyD88 mice and WT mice at 24 hours post CLP surgery. Homogenates were subjected to Western blotting with simultaneous detection of actin and occludin (A) or claudin-5 (B) using differentially labelled secondary antibodies. Sample conditions and sizes were as indicated in the blot image annotation (A, B). Band identity was confirmed using lysates from transfected recombinant cells. Band intensity was quantified using densitometry for occludin (C) and claudin-5 (D). n.s., p>0.1, no significant effect of genotype (t-test).

Alterations in the expression and/or localization of the tight junction proteins occludin and claudin-5 have been associated with BBB permeability [78]. In order to evaluate whether the lack of endothelial MyD88 was associated with alterations in the expression and/or localization of these key BBB factors, we performed immunofluorescence staining of BBB proteins in cortical sections and Western blotting in homogenized whole brain tissue from WT and ΔEndoMyD88 mice subjected to CLP. Immunofluorescence data are depicted in Fig 5 and Western blotting data with densitometry quantification are depicted in Fig 6. No significant effects of genotype were observed. However, as we did not assess the effect of CLP per se, we cannot rule out the possibility of genotype-CLP interactions which may have affected tight junction protein expression in our model.

The vagus nerve is an important conduit of information between the visceral organs and the CNS. Sensory fibers from the vagus innervate the peritoneum and provide a potential anatomical basis for rapid neuronal sensing of peritoneal infection [38, 79–81]. The vagus nerve is required for the neuronal anti-inflammatory reflex, which modulates cytokine secretion during acute inflammation, and may be involved in additional CNS-mediated reflexes during systemic inflammation [38, 81–83]. This neuronal conduit may play a role in hippocampal gene expression in response to LPS injection [84, 85], but the role the vagus nerve in forebrain physiological responses to CLP has not been addressed.

**Fig 7:**
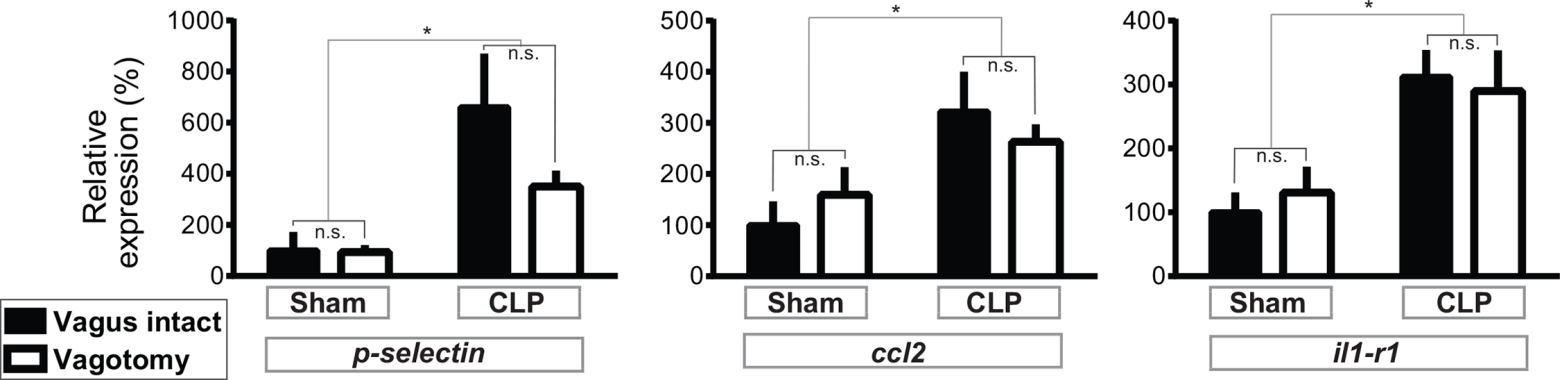
CNS transcriptional response to polymicrobial sepsis do not require an intact vagus nerve. WT mice were subjected to bilateral subdiaphragmatic vagotomy or control surgery, then subjected to CLP surgery or Sham laparotomy. Hippocampi were dissected 24 hours post-surgery and expression of indicated genes was analyzed by qPCR. Data were analyzed by 2-way ANOVA. *p<0.03, significant effect of CLP surgery; n.s., p>0.1, no significant effect of vagotomy and no significant interaction (2-way ANOVA); error bars represent standard error of the mean. Sample size, 5–6 per condition.

In order to determine whether an intact vagus nerve is required for transcriptional responses to CLP in the hippocampus, we combined CLP and bilateral subdiaphragmatic vagotomy, with the corresponding control surgeries, in male C57BL6/J mice aged 4 months old. Bilateral vagotomy did not significantly affect CLP-induced elevation of serum IL-6, as we have described previously in a rat CLP model [86]: serum concentrations (pg/mL, ± standard error of the mean) were 53±35 in sham-operated mice; 242±27 with vagotomy alone, 80934±14518 with CLP alone, and 106428±14517 with vagotomy and CLP. Vagotomy alone did not affect expression of *Selp*, *Ccl2* and *Il1r1* in the hippocampus. CLP-induced elevation of all three markers was observed and was similarly unaffected by vagotomy (Fig 7). The amplitude of CLP-induced transcription was generally lower for both vagotomized and vagus-intact mice in this experiment (compare with Fig 4), perhaps due to a neuroinflammatory effect of the more complex control surgeries. (This difference was not due to genetic differences, as conditional *myd88* mutant mice bred in-house were observed to be indistinguishable from Jackson-purchased C57BL6/J mice with respect to CLP-induced CNS transcription in separate experiments not shown.) These results suggest that in the case of polymicrobial sepsis, the vagus nerve is not required for transcriptional responses in the forebrain.

## Discussion

A number of elegant studies to date have demonstrated the role of TLR signaling and MyD88 in neurovascular activation and neuroinflammation during systemic infection. The utility of constitutive null mutant mouse alleles has been limited by the fact that genes in these pathways are required for cardinal features of systemic sepsis, such as the cytokine storm [58, 60, 62, 87, 88]. The absence of a conventional sepsis response complicates the interpretation of CNS sequelae. To address this issue, investigators have employed bone marrow transplantation to target mutations to bone-marrow derived hematopoietic cells (circulating myeloid and lymphoid cells) or radiation-resistant non-hematopoietic cells, including CNS-resident endothelial cells and neurons. Resulting chimeric mice were then treated with various endotoxins or cytokines. These studies must be interpreted in light of recent reports that radiation required for bone marrow transplantation can affect the BBB and influence the trafficking of microglia-like myeloid cells into the CNS [89, 90]; nevertheless, important insights have emerged. TLR4 in radiation-resistant cells was shown to be required for recruitment of leukocytes to CNS blood vessels (a classic manifestation of endothelial inflammation) following intra-CNS or intraperitoneal injection of LPS; transgenic expression of TLR4 on endothelial cells alone was sufficient for leukocyte recruitment in response to low-dose systemic LPS administration [28, 48]. These studies also demonstrated a role for MyD88 in leukocyte recruitment using constitutive null mutants. The transcriptional CNS response to LPS was similarly shown to require radiation-resistant, but not hematopoietic TLR4. In fact, hematopoietic TLR4 was sufficient for LPS-cytokine induction in the circulation, but not in the CNS [49].

In an additional study, reciprocal bone marrow transplantation was performed using MyD88 constitutive null mutant mice and wild-type controls [29]. Using intravenous IL-1 administration to trigger MyD88-dependent systemic inflammation, the authors concluded that MyD88 in radiation-resistant cells was required for the induction of prostaglandin expression in endothelial cells, for neuronal activation and for the subsequent engagement of specific CNS-mediated reflexes. In fact, a number of studies using bone marrow chimeras and other models have led to the conclusion that CNS sensing of systemic inflammation can occur even in the absence of a normal peripheral innate immune response [28, 48, 49, 51]. One exception is a recent study reporting that LPS-induced anorexia required hematopoietic MyD88, but not cerebrovascular or neuronal MyD88 [91]. Importantly, the CNS effects of systemic inflammation may be highly dependent on the specific preparation, species and developmental state under analysis [92]. Taken together, the body of work described above has been interpreted to support of the hypothesis that MyD88-dependent TLR signaling in CNS endothelial cells is an important mediator of neurovascular inflammation and downstream effects of CLP in the CNS.

Here, we used a conditional gene targeting approach to specifically address the role of MyD88 in endothelial cells during polymicrobial sepsis. Surprisingly, we did not observe significant induction of gene expression for tumor necrosis factor α nor interleukin 1β in the hippocampus during CLP sepsis, contrary to previous reports [93]; this discrepancy may be related to differences in sepsis severity, which can vary depending on multiple experimental parameters [54]; on the anatomical sampling; or on differences in assay detection limit (the expression of cytokines generally being much lower in the CNS than in many other tissues, even during systemic infection). However, we demonstrated a robust induction of several CNS-intrinsic proinflammatory factors in the hippocampus following CLP, which did not require endothelial MyD88. These results are consistent with two possible models. First, during a complex polymicrobial infection, MyD88-independent signals, such as tumor necrosis factor α, may be sufficient to stimulate neurovascular inflammation and BBB breakdown. These neurovascular events could then affect CNS gene expression. Alternatively, the loss of endothelial MyD88 could abrogate neurovascular inflammation, however CNS transcriptional responses could be mediated by an alternative anatomical pathway, such as a purely neuronal mechanism.

In order to distinguish between these two models, we employed a powerful imaging method, transcranial two-photon imaging, the results suggesting that endothelial MyD88 is not required for BBB deterioration during sepsis. While our study thus cannot directly address the role of endothelial inflammation in the transcriptional effects of CLP observed, our results suggest that redundant molecular signals, including MyD88-independent signals, can trigger neurovascular inflammation and BBB breakdown during polymicrobial sepsis. This leaves open the possibility that these events are important for acute and chronic effects of systemic infection on CNS function. This conclusion contrasts with studies cited above, where endothelial TLR signaling was proposed to be required for the effects of LPS on neurovascular dynamics and CNS transcription. This distinction may be related to the increased cell-type specify of the Cre-Lox system, as compared with bone marrow chimera approaches.

However, the more critical distinction is likely the fact that our study employed a live infection, as opposed to administration of LPS or IL-1, which more specifically engage MyD88-dependent signal transduction mechanisms. A polymicrobial infection presents a much more complex set of PAMPs and triggers a distinct systemic cytokine response. The pharmacology, dynamics and dosing of innate immune receptor stimulation are additional critical differences between experimental endotoxemia and CLP sepsis. It is perhaps not surprising that CLP sepsis could mediate conserved physiological responses through both MyD88-dependent and–independent mechanisms. In fact, even in the case of LPS administration, innate immune responses can be observed in TLR4 null mutant mice, depending on the level of LPS purity [49]. The relative contribution of MyD88-dependent signaling in CNS inflammation could be further elucidated by comparing neuroinflammatory responses in CLP and endotoxemia model in constitutive MyD88 null mutant mice.

One study demonstrated that intracerebroventricular LPS, but not low-dose systemic LPS, could compromise the BBB and induce CNS transcription of inflammatory markers [92]. We additionally sought to address the role of non-endothelial structures in CNS-induced gene expression during CLP sepsis. Importantly, neurovascular activation and BBB degradation can be a result of inflammation originating within the CNS; for example, direct neuronal sensing of peritoneal infection could lead to CNS-intrinsic inflammation, with BBB degradation occurring secondarily, as in some cases of epilepsy [94]. Transduction of neuronal signals from the visceral organs to the CNS via the vagus nerve is in fact a major mechanism of CNS engagement during sickness and injury. Previous reports have demonstrated a requirement for an intact vagus for the induction of CNS gene expression and CNS-mediated physiological responses following systemic LPS administration, although this dependence may vary with the LPS dose [81, 83–85, 95, 96]. One of these neuronal reflexes regulates the magnitude of the innate immune response itself [38]. Interestingly, although neuronal processes can directly respond to IL-1 and bacterial products [97, 98], neuronal MyD88 is not required for IL-1-induced sickness behavior [99], perhaps reflecting distinct MyD88-independent signal transduction mechanisms in neurons [76]. We found that sepsis-induced gene expression in the hippocampus did not require an intact vagus nerve. Importantly, we did not perform a combined manipulation to simultaneously abrogate the vagus nerve and endothelial MyD88 signaling. As a future study, it would be informative to perform this experiment in order to determine whether the simultaneous abrogation of both pathways would interrupt redundant pathways to block infection-induced CNS inflammation. Based on our results and the works cited above, it is likely that CNS inflammation would still be observed in this situation.

We propose that a non-vagal neuroanatomical circuit contributes to the observed MyD88-independent effects of CLP in the CNS. In fact, it is increasingly clear that multiple types of neurons have the capacity to directly sense and respond to bacterial products and cytokines via mechanisms which are distinct from canonical MyD88-dependent innate immune signaling described in myeloid and other cells. For example, IL-1 activates the p38 mitogen-activated protein kinase pathway in hippocampal neurons, whereas this same cytokine activates the proinflammatory nuclear factor-κB pathway in adjacent astrocytes [100]. In the hypothalamus, neuronal responses to peripheral inflammatory responses have been described [101], although such neuronal responses may not necessarily drive non-neuronal MyD88-dependent inflammation. Of particular relevance to the present study, nociceptive neurons of the dorsal root ganglia, which have recently been shown to directly transduce extracellular bacterial products into electrical activity, could mediate CNS sensing of peritoneal infection [102]. According to this model, bacterial products present in the peritoneum during the initiation of CLP sepsis would activate local nociceptive fibers, which would then transmit electrophysiological signals to the forebrain via the dorsal column and CNS relays. Additional important anatomical and cellular mediators of blood-to-brain signaling during CLP sepsis include the cerebroventricular organs [103] and perivascular macrophages. The interface between the blood and the cerebrospinal fluid is another important potential site for interaction between the CNS and the circulating immune system during systemic inflammation [25, 104].

Taken together with previous work, our results suggest that BBB permeabilization and CNS transcriptional activation can occur through multiple robust and redundant cellular and molecular pathways. Nuclear factor-κB signaling is one additional pathway that has been shown to be important for endothelial inflammation during sepsis [105–107] and may be activated downstream of MyD88-dependent or through MyD88-independent mechanisms. In preliminary work, we have observed intact CNS responses to CLP in mice with suppression of nuclear factor-κB signaling in endothelial cells [107] and even in constitutive MyD88 null mutant mice.

BBB degradation and CNS inflammation have been well studied with respect to their potential negative consequences for neurological function. However, the robust and redundant nature of CNS activation during sepsis raises the question of whether these responses play a role in host defense as well. Microvascular permeabilization during sepsis is generally thought of as pathological, but it may also be required to recruit and mobilize adequate immune-mediated and CNS-mediated defenses to prevent infection of the CNS and to regulate physiological responses to systemic infection. A more complete mechanistic understanding of neurovascular and neuronal physiology during polymicrobial sepsis will be invaluable in the design of therapeutic approaches for the devastating effects of sepsis.

## Materials and Methods

### Ethics statement regarding animal experiments

This study was carried out in strict accordance with recommendations in the Guide for the Care and Use of Laboratory Animals of the National Institutes of Health. The protocol was approved by the Institutional Animal Care and Use Committee of the Feinstein Institute for Medical Research (approval number: 2009–048). All surgery and *in vivo* imaging was performed under anesthesia with continuous monitoring of depth using multiple physiological parameters. Ketamine/xylazine and/or isoflurane were applied using doses recommended by veterinary guides for this purpose. Post- surgically, mice were closely monitored and immediately euthanized if moribund. Euthanasia was accomplished using recommended humane methods (pentobarbital overdose followed by cervical dislocation).

### Mouse genetics

To generate ΔEndoMyD88 mice, founder breeder mice from two lines were obtained and interbred: male and female *myd88*^Flox/Flox^ conditional mutant mice (Jackson stock #008888, formal designation: B6.129P2(SJL)-*Myd88*^*tm1Defr*^/J; Mouse Genome Informatics ID: MGI:3809600); and male *tek-cre*^Cre/0^ (Jackson stock # 004128, formal designation B6.Cg-Tg(Tek-cre)12Flv/J; MGI ID: 2136412). In parallel, *myd88*^Flox/Flox^ mice were generated by interbreeding homozygous founders. For gene expression experiments, F1 *tek-cre*^Cre/0^, *myd88*^Flox/+^ male mice were bred with F1 *myd88*^Flox/Flox^ female mice to generate F2 experimental *tek-cre*^Cre/0^, *myd88*^Flox/Flox^ and littermate control *tek-cre*^0/0^, *myd88*^Flox/Flox^ mice. For two-photon experiments, F2 *tek-cre*^Cre/0^, *myd88*^Flox/Flox^ male mice were bred with *myd88*^Flox/Flox^ female mice to generate experimental and control genotypes as above. For vagotomy experiments (Fig 5), C57BL/6J male mice (Jackson stock #000664) were used. For experiments on constitutive *myd88* null mice, mice were purchased from Jackson Laboratories (MGI ID:4421295). For experiments on GFP reporter mice, homozygous knock-in female mice (MGI ID: 3716464) of the *rosa26*^GFP/GFP^ genotype were crossed with *tek-cre*^Cre/0^ males to generate F1 experimental *tek-cre*^Cre/0^*; rosa26*^GFP/GFP^ and control *tek-cre*^0/0^*; rosa26*^GFP/GFP^ mice which were analyzed at 8 weeks of age.

### Mouse husbandry

All mouse lines were obtained from Jackson Laboratories under Specific Pathogen Free conditions and maintained in a conventional facility at the Feinstein Institute. For experiments, virgin male mice aged 3–5 months were used. For mice bred in-house, breeding was performed using 2–4 month-old females and 2–6 month old males. Trio harem breeding was maintained until pregnancy of newborn litter was observed, at which point one female was removed to a new cage until weaning. Chow provided was LabDiet 5001 (St. Louis, MO) *ad libitum*. Light cycle was 12 hours light, 12 hours dark. Cages were changed once weekly. Cage changes were not performed within 3 days of experimentation or litter birth. Mouse breeding and experimental manipulations were tracked using the Jackson Laboratories Colony Management System database. At 21 days of age, mice were separated by sex, marked using an ear numerating system and a minimal (<0.5 mm) tail biopsy was collected for genotyping. Mouse genotyping was performed using a gel-free high-throughput method as described below. For vagotomy experiments mice were shipped from Jackson Laboratories at 7 weeks of age and allowed to acclimate for 4–6 weeks prior to surgeries.

### Surgeries: General considerations

All surgeries were performed under aseptic conditions. A deep anesthetic plane was maintained at all times with regular monitoring of multiple physiological parameters including negative toe pinch reflex, eyelid movements, breathing rate. For CLP surgeries and initial preparation for intracranial imaging, temperature was monitored and maintained above 33°C; during imaging, temperature was not maintained so as to allow the normal fever response. Surgeries were initiated between 1200 and 1600 during the light portion of the circadian cycle.

### CLP, sham and vagotomy surgery

Anesthesia was induced using 3–4% isoflurane and maintained at 0.8%-2.5% in order to maintain anesthetic depth. CLP was performed according to a detailed protocol paper, with the protocol titrated to produce ‘mid-grade’ sepsis as defined in that work [108]. Briefly, the abdomen was shaved and disinfected with iodine. A small (1 cm) incision through the skin and peritoneal wall was performed. The cecum was exteriorized. Position of ligation was chosen at half the distance between distal pole and the base of the cecum (identified by the ileocecal valve). At this level, the cecum was ligated using silk suture and a double surgical knot. Two punctures were placed in the ligated portion, using a 22G needle. The cecum was then gently compressed with forceps until the patency of the puncture was verified by the extrusion of a small amount (< 1 mm^3^) of cecal content. The cecum was then placed back in the cavity, with care taken not to disturb the extruded material. The interior was sutured using nylon suture and the exterior wall was sutured using silk suture. Anesthesia was then withdrawn and 1 mL of 5% dextrose in sterile 0.9% NaCl was administered subcutaneously for fluid resuscitation. Mice were placed on a clean surgical pad in a clean cage and monitored closely until regaining righting reflex (typically 5–8 min). Duration between initial anesthesia and recovery was less than 25 minutes. For sham control surgeries, all steps were followed as above except that ligation and puncture were omitted. Mice were monitored every 8 hours following surgery and were euthanized if moribund (lack of righting reflex, inability to feed, immobility). Characteristic signs of sepsis (sickness behavior, low activity, dehydration, diarrhea, and bacteremia) were consistently observed in the CLP but not sham-operated mice. Under these conditions in our laboratory, 3-day mortality from CLP was approximately 40%. Sample sizes are indicated in figure legends.

For vagotomy experiments, vagotomy was performed under isoflurane anesthesia at the same time as CLP or control surgery. The subdiaphragmatic vagus nerve was exposed from the ventral aspect and severed. For control surgeries, the vagus nerve was gently exposed but not manipulated further. Food but not water was withheld in vagotomized and corresponding control mice during the 24 hours between surgery and dissection.

Within the 24-hour time window in this study, mortality prior to dissection or imaging was rare (<5%) in all CLP groups (including vagotomized mice), with no effect of genotype or vagotomy evident. Mortality was not observed in the sham-operated group.

### Imaging surgery & procedure

Imaging was performed using a method adapted from described protocols [67, 68, 109] 24 hours following CLP surgery, mice were anesthetized by intraperitoneal injection of ketamine (100 mg/kg) and xylazine (8 mg/kg). When anesthesia was achieved, mice were placed on a heating pad under a stereoscope. A 2 cm incision was made to expose the skull. Connective tissue was removed with application of a small amount of 1% hydrogen peroxide and abrasion with a scalpel. A custom-built metal plate with an elliptical hole of 8 mm length was then bonded to the skull using cyanoacrylate glue (Krazy Glue) applied to the rim of the hole, such that the center of the hole was at the coordinates of the primary somatosensory cortex (as determined relative to bregma using the Paxinos atlas). The top of the skull was thus elevated above the plane of the metal plate. Once the plate was fully bonded, the metal plate was affixed to a custom-built stage and a small section (approximately 500 μm^2^) of the skull was thinned using a drill fitted with a 700 μm micro bit (Fine Science Tools). Care was taken to minimize drill speed and take frequent pauses to prevent overheating. Powdered bone was evacuated with air puffs. Once the pial blood vessels were visible, a #15 microsurgical blade was used for further thinning using a scraping motion under a drop of sterile water. The skull was thus gently thinned to a thickness of less than 50 μm (as verified under two-photon microscopy). If the skull was ruptured or if a pial vessel burst during the process, the preparation was discarded.

Once the surgery was completed (typically less than 40 minutes), imaging was performed immediately. 100 μg of 2 MDa size dextran labelled with fluorescein isothiocyanate (Sigma-Aldrich catalog # 52471) in 50 μL saline was injected intravenously through the retro-orbital sinus. The preparation was placed immediately under the microscope objective for imaging over the thinned area. At this point, isoflurane anesthesia was initiated (0.5–1%) to maintain anesthetic plane. This combination of anesthesia modalities was chosen to minimize death of septic mice during the imaging session (under the conditions described, mice typically remained stable throughout the 60–90 minutes of imaging).

Imaging was performed using a customized Olympus FV1200 upright microscope and Olympus software. A reservoir for water immersion was created by drawing a circle on the skull plate using a hydrophobic PAP pen and placing a drop of distilled water over the skull. A 25X long working distance immersion objective (Catalog #XLPL25XWMP, Olympus) was then lowered over the imaging area and the pial vessels were brought into focus using brightfield or halogen illumination. At this point, the microscope was switched into laser scanning mode and 800 nm wavelength two-photon excitation was applied using a locked laser (Mai Tai DeepSee, Spectraphysics) at 3% maximal strength. Emission was collected using photomultiplier tubes outfitted with the following band pass filters: 502–547 nm (fluorescein) and 457–487 nm (Cascade Blue). Using the lowest wavelength filter, the z-position of the remaining bone was determined, as well as the thickness. Blood vessels were visualized using the fluorescein signal from the dextran. A focal plane 30–40 nm below the pia was chosen, containing small-to-medium sized vessels (and not containing large descending vessels). A second retro-orbital injection of 100 μg of 10 KDa sized Cascade Blue labelled dextran (Life Technologies catalog #D1976) was then administered and image acquisition was initiated. Photomultiplier tube sensitivity was adjusted such that peak signal at the center of a vessel was below saturation. No other adjustments were made to offset or other parameters, with the exception of a minor depth correction of laser strength. All laser and sensitivity settings were identical throughout a given imaging session. 30 optical sections spaced at 1 μm intervals (starting at 30–50 μm depth determined as above) were collected from top to bottom at four time points: 0, 15, 30 and 60 min (time refers to the initiation of image acquisition of a z-stack; each z-stack took approximately 15 minutes to acquire).

For preparation of final images as shown, maximal intensity projection were generated using ImageJ software. The only image parameter adjusted was false-color of each channel and the dynamic range of pixel display. All such parameters were applied equally to every time point within an imaging session, without signal saturation.

### Image analysis

Image quantification was performed on a Zen2 system (Zeiss, Thornwood) that generated a segmentation strategy to separate the mean intensity of the FITC and the Cascade Blue signal over the entire image and, for replication, also over randomly placed regions of interest (data not presented). Using the FITC signal as a mark of the vessel, the program measured Cascade Blue signal that did not overlap with the FITC signal. Individual Z-planes were analyzed, and, in another analysis, Z-stack images were collapsed into the single best plane for analysis. These results were comparable (data not presented). The area of each signal was measured and a mean intensity for the Cascade Blue signal resulted. Table 1 subtraction of the mean intensity value at 0 and 30 min for each mouse, as calculated from images obtained immediately after dye injection and 30 minutes post injection. Because the sample size in each group was limited we present the mean intensity data (grey values and unit-less; and called an extravasation index) for an entire image plane. The results in this small sample support the lack of effect for the MYD88 intervention.

### Necropsy and sample collection

For sample collection (for serum analyses and mRNA extraction), mice were euthanized with an overdose of Euthasol (pentobarbital sodium and phenytoin sodium) (0.3 mL intraperitoneal injection). When deeply anesthetized but prior to cardiac arrest, 100 μL serum was collected by cardiac puncture using Gold Microtainer tubes (Beckton Dickinson) and stored on ice. Mice were then immediately transcardially perfused with 5–10 mL phosphate-buffered saline. Perfusion was verified by clear perfusate and evacuation of blood from major cerebral vessels. Brain was then dissected and the entire right hippocampus removed and immediately homogenized in 500 μL Trizol reagent (Life Technologies) and stored at room temperature until further processing.

### Genotyping procedure

Gel-free high-throughput PCR genotyping was performed using a modified published method [110]. Tail biopsies were digested in 8-well strip tubes with 0.05 U proteinase K (03115887001, Roche) in 100 μL of DirectPCR Lysis Reagent (402-E, Viagen Biotech). Heat inactivation at 85°C for 45 min was performed prior to use. Genomic DNA from CNS microvessels (purification described below) was prepared from Trizol homogenate according to the manufacturer’s standard protocol (Life Technologies).

### PCR procedure

For all assays (genotyping and gene expression), with the exception of the *Ccl2* assay, 6 μL PCRs were performed consisting of 3 μL SYBR Green I Master PCR mix (Roche), 1 μL template and 200 nM each of 2 primers. Primer design is described below. For *Ccl2* assays, 6 μL PCRs were performed consisting of 3 μL ProbesMaster PCR mix (Roche), 1 μL template and 0.3 μL of a commercially available primer/probe cocktail (4331182, TaqMan gene expression assays, Life Technologies). Cycling was performed in 384-well plates on a LightCycler 480 instrument controlled using LightCycler 480 Software v.1.5 with the following thermal conditions: 95°C for 5 min, followed by 40 cycles of 95°C for 10 s and 60°C for 30 s; followed by a melting curve analysis (95°C for 5 s; 65°C for 1 min; ramping up to 97°C at 0.11°C/s). For the *myd88_B* assay, thermal conditions were: 95°C for 5 min, followed by 40 cycles of 95°C for 10 s, 66°C for 30 s and 72°C for 45 s; followed by a melting curve analysis. Optical data was collected using the FAM filter (excitation at 465 nm, emission at 510 nm) at the conclusion of each annealing step (or 5 acquisitions per second during melting curve analysis).

### PCR assay development

Primer sequences are reported in Table 2. Prior to primer design, target sequences were obtained by direct sequencing or by queries in the UCSC Genome Browser. Assays were designed *de novo* using Primer3Plus software with standard conditions for SYBR qPCR, except for two assays. In brief, target primer melting temperature was 60°C; optimal length was 20 bp; primer GC proportion was 30–80%; amplicons length was 70–120 bp; and Primer3 algorithms were applied to prevent mispriming. *Ccl2* assay was obtained commercially, as noted above. The *myd88_B* assay was adapted from a protocol obtained from Jackson Labs. Melting curve analysis was performed to confirm that the final assay generated a single melting peak associated with a homogenous amplicons pool.

**Table 2:** PCR primers & assays.

Target name	Assay type	Forward primer sequence	Reverse primer sequence	Notes
*k17*	Genomic reference	ggcgagagcagagtgtggat	aagtcggcaggcacaggag	Amplifies any mouse genomic DNA
*myd88_A*	Genomic	cttcctcccagatgaaatcc	tgggaataatggcagtcctc	Amplifies the wild-type allele only (not the conditional nor recombined allele)
*myd88_B*	Genomic	gttgtgtgtgtccgaccgt	gtcagaaacaaccaccaccatgc	Amplifies the wild-type or conditional allele (not the recombined allele)
*cre*	Genomic	acatttgggccagctaaacat	cggcatcaacgttttctttt	Generic assay amplifies any allele bearing a *cre* cassette
*mpolr2*	Gene expression reference	aagtcggcaggcacaggag	ggcgagagcagagtgtggat	Gene for murine RNA Polymerase II; widely used reference gene for relative gene expression quantification; exon spanning
*il1r1*	Gene expression	ggagaaatgtcgctggatgt	tttgtgttgttcacggttcg	Not exon spanning
*selp*	Gene expression	atcgagaccattgggagcta	acactctggcccatagaagc	Exon spanning
*tnf*	Gene expression	Proprietary commercial assay (Applied Biosystems, Catalog #4331182, TaqMan gene expression assays)		Exon spanning
*il1b*	Gene expression	Proprietary commercial assay (Applied Biosystems, Catalog #434228, TaqMan gene expression assays)		Exon spanning
*ccl2*	Gene expression	Proprietary commercial assay (Applied Biosystems, Catalog #443258, TaqMan gene expression assays)		Exon spanning

### PCR analysis

qPCR analysis was based on determination of threshold cycle (Ct) using the internal high confidence maximal second derivative algorithm of the LightCycler software. No reference dye was used; fluorescence was normalized to a baseline measurement using standard internal parameters. All amplification curves were visually inspected to ensure that reported Ct was not artifactual. Duplicates and triplicates were averaged prior to further analysis. Wide-range standard curves were constructed using serial dilution of inflamed spleen cDNA or genomic DNA as appropriate for the assay. Acceptable assays demonstrated linear amplification over a wide dynamic range with efficiency > 1.9 fluorescence increase per cycle in the linear range. For additional assay-specific information, see Table 2.

For tail genotyping, standard curves and appropriate negative controls were used to determine appropriate Ct cutoffs for positive amplification. In general, Ct < 25 cycles was considered positive and > 32 cycles was considered negative. A genomic reference assay (*k17*) was performed on all samples to confirm sample quality.

For qPCR of microvessel DNA and cDNA, the advanced relative quantification module of the LightCycler software was used to calculate relative fold abundance using a reference target (*k17* for microvessel DNA, *mPol* for gene expression). Standard curves were used to adjust calculation of fold abundance. Fold abundance was normalized to the mean fold abundance in the corresponding control condition.

### Serum analysis

Sera were processed within 3 hours of collection by centrifugation for 6 min at 2000 g at room temperature and collection of supernatant, which was stored at -80°C until further analysis. For determination of cytokine and chemokine concentrations, serum samples were thawed, diluted 5-fold and immediately processed using the Ultra-Sensitive 7-Plex Mouse Proinflammatory Cytokines kit on a Sector Imager 2400 instrument (MesoScale Discovery) according to the manufacturer’s instructions. Data were quantified by fitting to a standard curve using MesoScale Workbench software using default parameters.

### RNA extraction

RNA was extracted from Trizol (collected as above) using the manufacturer’s protocol (Life technologies) with the exception that 350 μL ethanol was substituted for isopropanol for the precipitation step. Resulting RNA was processed using the DNAFree kit (Life Technologies) and approximately 5 μg was used as a template for cDNA transcription using poly(A) priming and the Superscript RTIII kit (Life Technologies).

### CNS microvessel purification

Purification of CNS microvessels was performed by adapting a published density gradient method [111]. Briefly: one murine brain was used to generate one microvessel sample. Brains were removed, mechanically macerated to a size < 1 mm^3^ and digested for 75 min at 37°C with shaking at 250 RPM in the following: 3.3 mL DMEM media (Life Technologies) with 2.5 mg collagenase 2 (Worthington, LS004174) and 142 U DNAse I (Worthington, LS002058). Cells were recovered, resuspended in 8 mL 20% bovine serum albumin (Roche) and centrifuged at 1000 g for 20 min. Resulting pellet was then digested for 1 h at 37°C with shaking at 200 RPM in 2.5 mL DMEM with 1.7 mg collagenase-dispase (Roche, 1097–113) and 160 μg DNAse I. Cells were recovered, resuspended in 1 mL DMEM and layered on top of an isotonic 33% Percoll (Amersham, 17-0891-02) solution. Following centrifugation without deceleration at 1000 g for 10 min, the interphase containing the microvessels was collected. Cells were recovered. 10% were adhered to a glass slide using a cytospin apparatus and imaged using Numarksi brightfield optics. The remainder of the cells were homogenized in Trizol and processed as above.

### Histology

For GFP immunofluorescence, fresh-frozen brains were sectioned on a cryostat instrument at a thickness of 20 μm, thaw-mounted onto slides and stored at -80°C. Slides were then dried at room temperature, fixed for 20 min in freshly prepared cold 4% paraformaldehyde and stained with a chicken anti-GFP IgY antibody (Aves Labs) diluted 1:1000 and an Alexa Fluor 488-labelled secondary anti-chicken IgY (Life Technologies) diluted 1:400 in 1% bovine serum albumin in phosphate-buffered saline. Slides were imaged under epifluorescence illumination with a GFP filter set (Zeiss). For tight junction histology, hemi brains were frozen in powdered dry ice and stored in -80C. They were mounted with OCT and cut into 14um sections and stored in -80C till use. Sections were fixed in 95% ethanol in -20C for 30 min, then 1 min in -20C acetone. They were blocked with 1% BSA/PBS-T for one hour at room temperature, then incubated with primary antibody over night at 4C. We used the following primary antibodies: anti-claudin-5 (1:500, Invitrogen 341600) and anti-occludin (1:500, Invitrogen), with anti-CD31 (1:500, BD Pharmingen 557355) to identify vessels. Sections were washed 3 times in PBS-T, then incubated with secondary antibodies (AF488-anti-rabbit and AF594-anti-rat, Invitrogen, 1:400). Slides were then washed 3 times in PBS-T, and stained with DAPI (1ug/ml, in PBS) for 5 min. They were washed in PBS 3 times before mounting with coverslip, using DAKO Fluorescence mounting medium. An overview was acquired at 20x, and high resolution pictures were obtained using the Apotome with 40x objective. Staining and image acquisition were performed by an investigator blind to experimental condition.

### Western blotting

Hemibrains were homogenized in 10 volumes of ice cold RIPA buffer (ThermoFisher) containing protease inhibitors (Pierce) by pulsing using a homogenizer (ThermoFisher) for 5 seconds. The samples were then sonicated for 30 seconds (Misonix Sonicator) and centrifuged for 20 min at 18,000g. Supernatant was removed and protein content was quantified with a BCA protein assay kit (Pierce) according to manufacturer’s instructions. For detection of tight junction proteins, 15 μg of total protein was loaded into each well of a Novex 4–12% Bis-Tris gel system (Invitrogen). Gels were electrophoresed for 60 minutes at 200V with MOPS running buffer and subsequently proteins were transferred to Immoblot PDVF membrane at 40V for 2.5 hours. Following an one hour blocking step with 5% non-fat dry milk in PBS containing 0.1% tween for 1 hour at room temperature the blot was overnight incubated at 4C with primary antibodies diluted in 3% BSA/PBS (anti-claudin-5 (1:1000, Invitrogen), anti-occludin (1:1000, Invitrogen), anti-actin (1:1000, Abcam). On the second day the blot was washed 3 times for 15 minutes in PBS+0.1%tween and incubated with the secondary antibody prepared in 3% non-fat dry milk in PBS + 0.1% tween. Secondary antibody (680-DaMouse, 800-DaRabbit, Odyssey) was added at 1:5000 and incubated for 1 hr at RT. Following four washing steps Image acquisition was performed with an Odyssey Imaging System and proteins were quantified based on the densitometry of western blot bands.

### Statistical analysis

GraphPad Prism software was used for all analyses. Parametric analysis assuming Gaussian distribution was applied. For analyses with one factor, t-tests were performed.

For 2-factor analyses, 2-way ANOVA without matching was performed in order to simultaneously assess the effect and interaction of two factors (genotype and surgery for ΔEndoMyD88 experiments; sepsis condition and vagus condition for vagotomy experiments). Differences were considered significant if p < 0.05. Bonferroni post tests and non-parametric Mann-Whitney U tests were performed comparing every possible combination to confirm results of 2-way ANOVA.
